# Development of a vitrification method for preserving human myoblast cell sheets for myocardial regeneration therapy

**DOI:** 10.1186/s12896-018-0467-5

**Published:** 2018-09-10

**Authors:** Hirotatsu Ohkawara, Shigeru Miyagawa, Satsuki Fukushima, Shin Yajima, Atsuhiro Saito, Hiroshi Nagashima, Yoshiki Sawa

**Affiliations:** 1 Department of Cardiovascular Surgery, Osaka University Graduate School of Medicine, Suita, Osaka, Japan; 2 Laboratory of Developmental Engineering, Department of Life Sciences, School of Agriculture, Meiji University, Tama-ku, Kawasaki, Kanagawa Japan

**Keywords:** Cell sheet preservation, Vitrification, Skeletal myoblast, Scaffold-free construct, Cryopreservation, Tissue engineering, Functional recovery, Heart failure, Myocardial infarction, Animal model

## Abstract

**Background:**

Tissue-engineered cardiac constructs have potential in the functional recovery of heart failure; however, the preservation of these constructs is crucial for the development and widespread application of this treatment. We hypothesized that tissue-engineered skeletal myoblast (SMB) constructs may be preserved by vitrification to conserve biological function and structure.

**Results:**

Scaffold-free cardiac cell-sheet constructs were prepared from SMBs and immersed in a vitrification solution containing ethylene glycol, sucrose, and carboxyl poly-l-lysine. The cell sheet was wrapped in a thin film and frozen rapidly above liquid nitrogen to achieve vitrification (vitrification group, *n* = 8); fresh, untreated SMB sheets (fresh group, n = 8) were used as the control. The cryopreserved SMB sheets were thawed at 2 days, 1 week, 1 month, and 3 months after cryopreservation for assessment. Thawed, cryopreserved SMB sheets were transplanted into rat hearts in a myocardial infarction nude rat model, and their effects on cardiac function were evaluated. Cell viability in the cardiac constructs of the vitrification group was comparable to that of the fresh group, independent of the period of cryopreservation (*p* > 0.05)*.* The structures of the cell-sheet constructs, including cell-cell junctions such as desmosomes, extracellular matrix, and cell membranes, were maintained in the vitrification group for 3 months. The expression of cytokine genes and extracellular matrix proteins (fibronectin, collagen I, N-cadherin, and integrin α5) showed similar levels in the vitrification and fresh groups. Moreover, in an in vivo experiment, the ejection fraction was significantly improved in animals treated with the fresh or cryopreserved constructs as compared to that in the sham-treated group (*p* < 0.05).

**Conclusions:**

Overall, these results show that the vitrification method proposed here preserves the functionality and structure of scaffold-free cardiac cell-sheet constructs using human SMBs after thawing, suggesting the potential clinical application of this method in cell-sheet therapy.

## Background

Regenerative therapy using skeletal myoblast (SMB) sheets has been used experimentally to treat patients with heart failure, and its safety, feasibility, and potential efficacy have been demonstrated in clinical studies [1–5]. However, the application of regenerative therapy in the clinical setting is limited, owing to the need for an in-hospital cell processing center with an aseptic environment and the time-consuming protocols for preparing cell constructs. A resource stock of cell sheets for implantation would thus facilitate the wide application and industrialization of tissue implantation therapy. Nevertheless, a crucial challenge for the widespread adoption of cell sheet implantation is the development of an optimized method for the preservation of cell sheets that maintains their viability and regenerative function.

One established technique for cell preservation is the slow-freezing method using dimethyl sulfoxide (DMSO) [6, 7]. As temperature decreases, extracellular water freezes producing high osmotic pressure outside the cell, leading to dehydration and shrinkage. Because this osmotic pressure causes water molecules to move out of and freeze outside cells, intracellular ice formation is efficiently prevented. However, if the temperature decreases too rapidly, water molecules may not move outside the cells quickly enough, potentially leading to the formation of ice crystals inside the cells, damaging the cell membranes [8–10].

In contrast, the high concentration of cryoprotectant and extremely fast cooling rates in vitrification provide no time for the intra- and extracellular water to form ice crystals [11, 12]. Vitrification is applied to the preservation of many cell lines. According to previous reports, the function of tissues such as blood vessels is maintained after vitrification [13, 14]. In particular, vitrification is commonly used for preserving embryos in the clinical setting [15, 16]. Moreover, chondrocyte sheets reportedly maintain high viability after vitrification, which has thus been suggested for clinical use [17]. Therefore, in the present study, we evaluated whether vitrification could be used to preserve skeletal cell sheets in an implantable shape and with high potential to induce cytokine paracrine effects.

## Results

### Cell sheet shape before and after cryopreservation

**Fig. 1:**
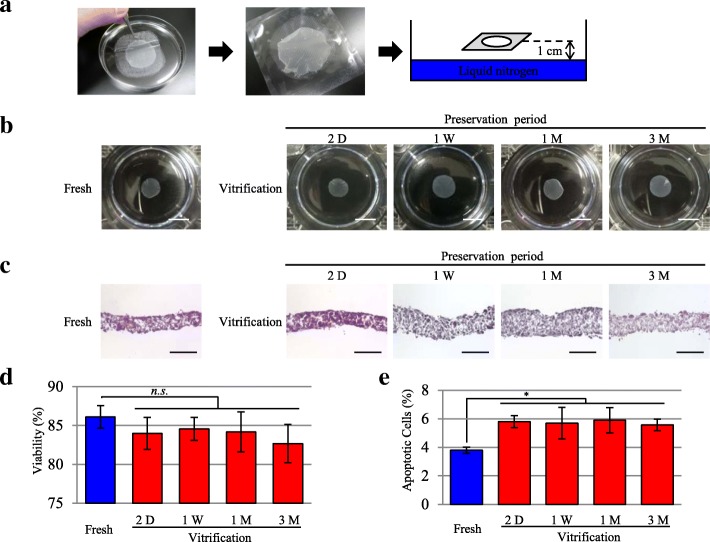
Effects of vitrification on cell shape and viability. **a** Vitrification method. The cell-sheet construct was immersed in the protective vehicle containing ethylene glycol, sucrose, and carboxyl poly-l-lysine; wrapped in a thin film using the mesh as a substrate for the cell sheet; and frozen above liquid nitrogen to achieve vitrification. **b** Cell sheet shape before and after cryopreservation. A representative image of the fresh group was obtained before cryopreservation. The cryopreserved cell-sheet constructs of the vitrification group were thawed at 2 days (D), 1 week (W), 1 month (M), and 3 months for assessment. Scale bar, 1 cm. **c** HE staining of the cell sheet. A representative image of the fresh group was obtained before cryopreservation. The cryopreserved cell-sheet constructs of the vitrification group were thawed at 2 days, 1 week, 1 month, and 3 months for assessment. Scale bar, 100 μm. **d** Cell viability in the cell sheet. The fresh group was assessed before cryopreservation. The cryopreserved cell-sheet constructs of the vitrification group were thawed at 2 days, 1 week, 1 month, and 3 months to evaluate cell viability. Results are expressed as the means ± SD (*n* = 8 independent experiments). **e** Apoptotic cells in the cell sheet. Frozen sections were immunostained with Annexin 5. The ratio of Annexin 5 (+) to all cells was calculated. The fresh group was assessed before cryopreservation. The cryopreserved cell-sheet constructs of the vitrification group were thawed at 2 days, 1 week, 1 month, and 3 months to evaluate apoptosis cell numbers. Results are expressed as the means ± SD (n = 8 independent experiments); *p < 0.05

The vitrification method is illustrated in Fig. 1a. The shape of the cell sheets in both the fresh group and the vitrification group was circular and filmy, without notable differences between the groups. This shape was maintained for at least 3 months in the vitrification group (Fig. 1b).

### Hematoxylin and eosin (HE) staining of the cell sheet

HE staining demonstrated that both the fresh group and the vitrification group had a high cell density in the cell sheets, with a rich extracellular matrix (ECM). The cell density was maintained in the vitrification group for at least 3 months after preservation (Fig. 1c).

### Cell viability in the cell sheet

The viability assay revealed that the viability of cells in the cell sheet in the vitrification group was comparable to that in the fresh group. Specifically, the fresh group showed 86.1 ± 1.4% viability, whereas at 2 days, 1 week, 1 month, and 3 months, the viability in the vitrification group was 84.0 ± 2.1%, 84.6 ± 1.5%, 84.2 ± 2.6%, and 82.7 ± 2.5%, respectively (*p* > 0.05). In addition, no significant difference was observed in the viability estimates between vitrification groups at different time points (Fig. 1d).

### Apoptosis analysis

The apoptosis analysis revealed that the number of apoptotic cells in the cell sheet was significantly higher in the vitrification group than in the fresh group. The fresh group showed 3.8 ± 0.2% non-viability, whereas at 2 days, 1 week, 1 month, and 3 months, the non-viability level in the vitrification group was 5.8 ± 0.4%, 5.7 ± 1.1%, 5.9 ± 0.9%, and 5.6 ± 0.4%, respectively (*p* < 0.05). There was no significant difference in the non-viability estimates between vitrification groups at different time points (Fig. 1e).

### Evaluation of cell-cell adhesion by transmission electron microscopy (TEM)

**Fig. 2:**
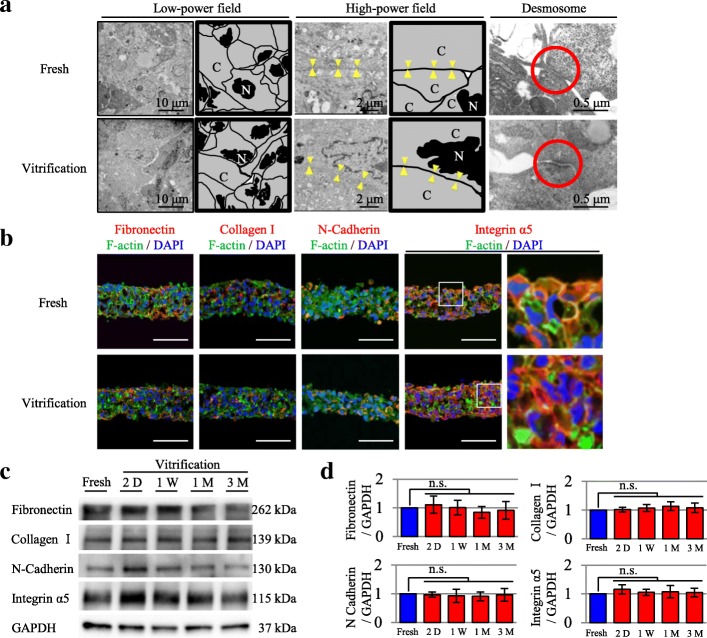
Effects of vitrification on cell-cell adhesion and ECM integrity. **a** Evaluation of cell-cell adhesion in the fresh and vitrification groups by TEM. The triangles show the structure of cell-cell junctions. The circles show cell-cell junction structures such as desmosomes. C, Cytoplasm. N, nucleus. **b** Evaluation of the ECM by immunohistochemistry. Immunohistochemistry revealed that in both the fresh and vitrification groups, fibronectin, N-cadherin, and integrin α5 were expressed on the basement membrane in myoblasts. In addition, collagen I was expressed in the ECM. Scale bar, 100 μm. **c**, **d** Evaluation of the ECM by western blotting. Western blotting demonstrated the expression of fibronectin, collagen I, N-cadherin, and integrin α5 in the fresh and vitrification groups. The fresh group was evaluated before cryopreservation. The cryopreserved cell-sheet constructs of the vitrification group were thawed at 2 days (D), 1 week (W), 1 month (M), and 3 months for assessment. The results are expressed as the means ± SD (*n* = 8 independent experiments)

TEM observations revealed that cell-cell junction structures, such as desmosomes, were maintained after preservation in the vitrification groups. There were no apparent differences in the structure of cell-cell junctions between these groups (Fig. 2a).

### Effects of cryopreservation on the ECM

Immunohistochemistry revealed that in both the fresh and vitrification groups, fibronectin, N-cadherin, and integrin α5 were expressed on the basement membrane in myoblasts (Fig. 2b). In addition, collagen I was expressed in the ECM (Fig. 2b). Immunostaining and western blotting demonstrated that the expression of fibronectin, collagen I, N-cadherin, and integrin α5 did not significantly differ between the fresh and vitrification groups (Fig. 2c, d).

### Mitochondrial structure and expression of mitochondria-associated genes

**Fig. 3:**
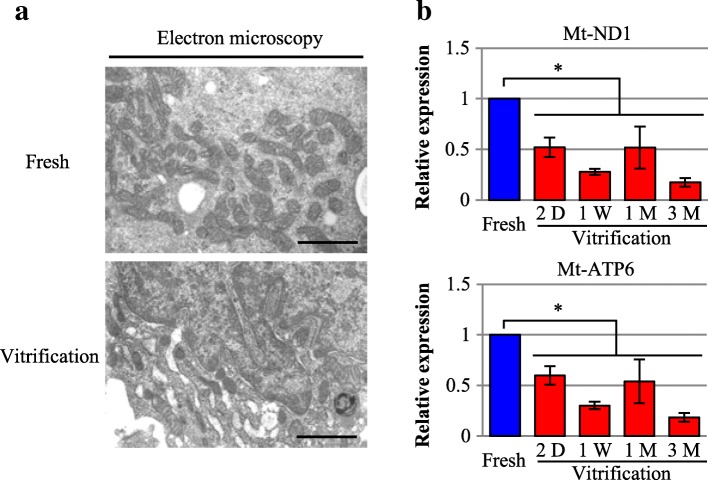
Effect of vitrification on mitochondrial function and structure. **a** Evaluation of mitochondrial structure in the fresh and vitrification groups by TEM. Scale bar, 1 μm. **b** Summary of RT-PCR results for the expression levels of the mitochondria-associated genes *mt-ND1* and *mt-ATP6* in the fresh and vitrification groups. The fresh group was evaluated before cryopreservation. The cryopreserved cell-sheet constructs of the vitrification group were thawed at 2 days (D), 1 week (W), 1 month (M), and 3 months for assessment. The results are expressed as the means ± SD (*n* = 8 independent experiments); *p < 0.01

Electron microscopy revealed that the shape of mitochondria and the structure of cristae were maintained in both the fresh and vitrification groups (Fig. 3a). Furthermore, quantitative reverse transcription polymerase chain reaction (qRT-PCR) demonstrated that the expression of the mitochondria-associated genes mitochondrial-encoded NADH dehydrogenase 1 (*mt*-*ND1*), and mitochondrial-encoded ATP synthase 6 (*mt*-*ATP6*) was significantly lower (*p* < 0.01) in the vitrification group than in the fresh group. However, succinate dehydrogenase complex, subunit A (*SDHA*) expression did not differ significantly between the groups (Fig. 3b).

### Cytokine expression

**Fig. 4:**
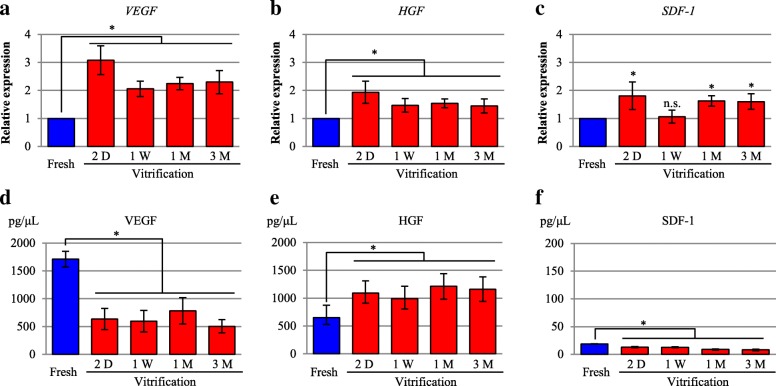
Effects of vitrification on cytokine expression in cell sheets. **a–c** qRT-PCR results for the expression of *VEGF*, *HGF*, and *SDF-1* in the fresh and vitrification groups. The fresh group was evaluated before cryopreservation. The cryopreserved cell-sheet constructs of the vitrification group were thawed at 2 days (D), 1 week (W), 1 month (M), and 3 months for assessment. The results are expressed as the means ± SD (*n* = 8 independent experiments); *p < 0.01. **d–f** ELISA results for the expression of VEGF, HGF, and SDF-1 in supernatants of the fresh and vitrification groups. The fresh group was evaluated before cryopreservation. The cryopreserved cell-sheet constructs of the vitrification group were thawed at 2 days, 1 week, 1 month, and 3 months for assessment. The results are expressed as the mean ± SDs (*n* = 8 independent experiments); *p < 0.01 vs. the fresh group

qRT-PCR analysis revealed that the levels of vascular endothelial growth factor (*VEGF*), hepatocyte growth factor (*HGF*), and stromal cell-derived factor-1 (*SDF1*) were upregulated in the vitrification group compared with those in the fresh group (Fig. 4a–c). However, based on enzyme-linked immunosorbent assay (ELISA), VEGF levels were significantly lower (*p* < 0.01) in the vitrification group (throughout the preservation period) than in the fresh group. In contrast, HGF expression was significantly upregulated (*p* < 0.01) in the vitrification group (throughout the preservation period) compared with that in the fresh group. The SDF-1 level was below the detection limit of the assay in both groups (Fig. 4d–f).

### Evaluation of cardiac performance after cell sheet implantation in the chronic ligation rat model

**Fig. 5:**
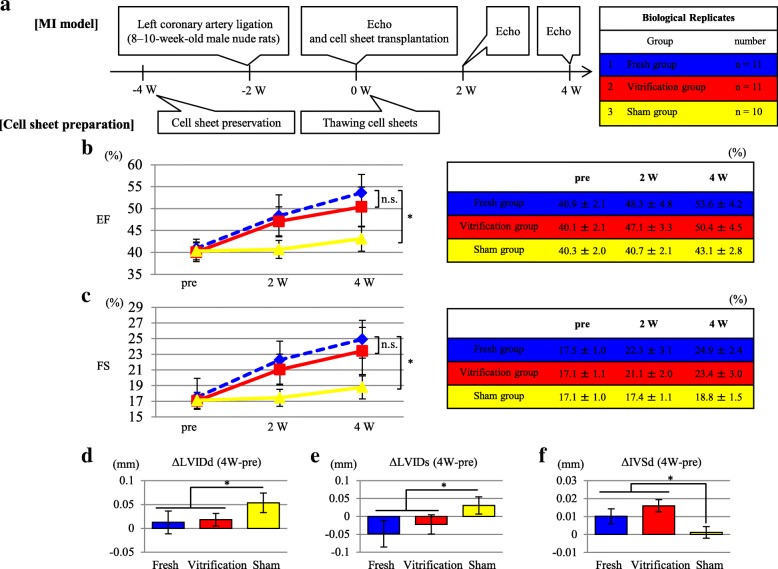
Effect of vitrification on the in vivo cardiac function of transplanted cell sheets. **a** Protocol for the analysis of cardiac function using an MI nude rat model. **b** Evaluation of EF before transplantation and at 2 and 4 weeks (W) after transplantation. Results are expressed as the means ± SD at each time point; *p < 0.05. **c** Evaluation of FS before transplantation and at 2 and 4 weeks after transplantation. Results are expressed as the means ± SD at each point; *p < 0.05. **d** Evaluation of the ΔLVDd before transplantation and at 4 weeks after transplantation. Results are expressed as the means ± SD; *p < 0.05. **e** Evaluation of the ΔLVDs before transplantation and at 4 weeks after transplantation. Results are expressed as the means ± SD; *p < 0.05. **f** Evaluation of the ΔIVSd before transplantation and at 4 weeks after transplantation. These results were evaluated in the 3 treatment groups: the fresh (cell sheets used without further treatment; *n* = 11), vitrification (n = 11), and sham operation groups (*n* = 10). Results are expressed as the means ± SD; *p < 0.05

**Fig. 6:**
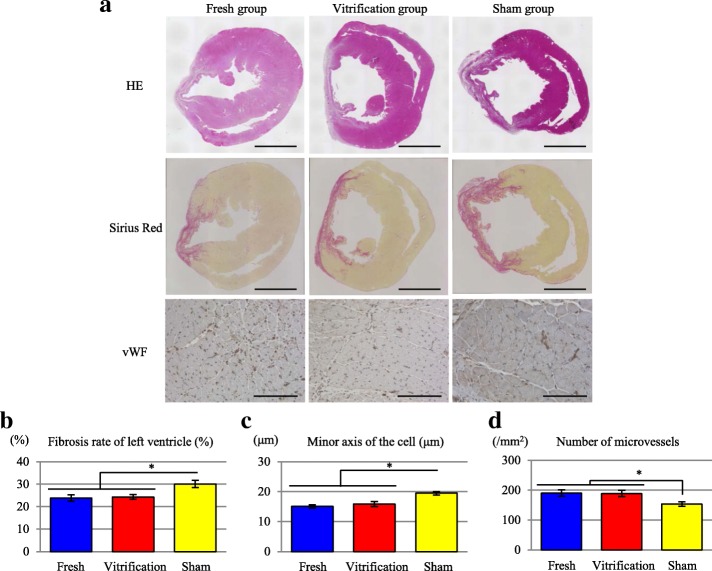
Effect of vitrification on in vivo cell sheet structure after transplantation. **a** Evaluation of microstructure after cell sheet implantation in the chronic ligation rat model. HE-stained sections for the fresh, vitrification, and sham groups at 4 weeks after transplantation. Sirius Red staining was used to evaluate the percent fibrosis of the chronic ligation rat model in the fresh, vitrification, and sham groups at 4 weeks after transplantation. The fibrotic area was stained red. Scale bar, 3 mm. The sections were immunostained with von Willebrand factor (vWF) antibody for evaluating the minor axis of the cells and the number of microvessels. Scale bar, 100 μm. **b** Percent fibrosis was calculated based on the fibrotic area in the left ventricle at 4 weeks after transplantation in the 3 treatment groups: the fresh (cell sheets used without further treatment; n = 11), vitrification (*n* = 11), and sham operation groups (*n* = 10) (%). Results are expressed as the means ± SD (*n* = 8 independent experiments); *p < 0.01. **c** The minor axis of the cells at the MI border area was measured at 4 weeks after transplantation in the 3 treatment groups (μm). Results are expressed as the means ± SD (*n* = 8 independent experiments); *p < 0.01. **d** The number of microvessels were measured at the MI border area at 4 weeks after transplantation in the 3 treatment groups (per mm^2^). Results are expressed as the means ± SD (*n* = 8 independent experiments); *p < 0.01

Ultrasonography revealed no improvement in the ejection fraction (EF) in the sham-treated group over time. However, EF was significantly improved in both the fresh and vitrification groups compared to that in the sham group throughout the study period. Similar improvements in the myocardial parameters fractional shortening (FS), difference in left ventricular (LV) end-diastolic dimension before and after treatment (ΔLVDd), difference in LV end-systolic dimension before and after treatment (ΔLVDs), and difference in interventricular septum dimension before and after treatment (ΔIVSd) were observed in the fresh and vitrification groups compared with those in the sham group (Fig. 5c–f). In addition to systolic function, the LV dimensions LVDd and LVDs were significantly lower in the vitrification and fresh groups than in the sham group (Fig. 5d, e). Sirius Red (SR) staining demonstrated that percent fibrosis was significantly lower in the vitrification (24.3% ± 1.1%) and fresh (23.8% ± 1.4%) groups than in the sham group (30.1% ± 1.7%; *p* < 0.01) (Fig. 6a, b). The minor axis of the cells at the myocardial infarction (MI) border area was significantly lower in the vitrification (15.9 ± 0.9%) and fresh (15.1 ± 0.6%) groups than in the sham group (19.5 ± 0.6%; *p* < 0.01) (Fig. 6a, c). In addition, the number of microvessels at the MI border area was significantly higher in the vitrification (189.2 ± 10.6/mm^2^) and fresh (190.4 ± 10.9/mm^2^) groups than in the sham group (153.9 ± 7.6/mm^2^; *p* < 0.01) (Fig. 6a, c).

## Discussion

The results of the present study demonstrate that vitrification is effective for the preservation of SMB cell sheets. Vitrified cell sheets maintained their rounded shape, similar to the shape observed in fresh cell sheets, for up to 3 months. Furthermore, cell viability in the cardiac constructs in the vitrification group was comparable to that in the fresh group, independent of the period of cryopreservation (*p* > 0.05)*.* In contrast, the fraction of apoptotic cells in the cell sheet was significantly higher in the vitrification group than in the fresh group. However, this was not sufficient to affect overall cell sheet viability.

Histological analyses, including HE staining and TEM, revealed that vitrified cells were dense and the ECM was intact with abundant, well-preserved desmosomes, similar to those in fresh cell sheets. Immunostaining and western blot analyses revealed that ECM-related proteins such as fibronectin, collagen I, and N-cadherin were expressed at the same levels in the basement membrane of the cells in the vitrification-preserved cell and fresh cell sheets. Integrin α5 was also expressed on the cell membranes of both types of cell sheets. Furthermore, mitochondrial cristae density and volume were similar in the vitrification-preserved and fresh cell sheets, whereas mitochondrial function appeared to be reduced after cryopreservation as determined by mitochondrial- related gene expression data. The potential for cytokine paracrine effects, a crucial parameter for clinical applications, was prominent in the cell sheets preserved by vitrification, and was comparable to that of the fresh cell sheets in the in vitro study. The same levels of functional improvement and histological recovery were observed in vivo after transplantation of the cell sheets in an MI rat model.

**Fig. 7:**
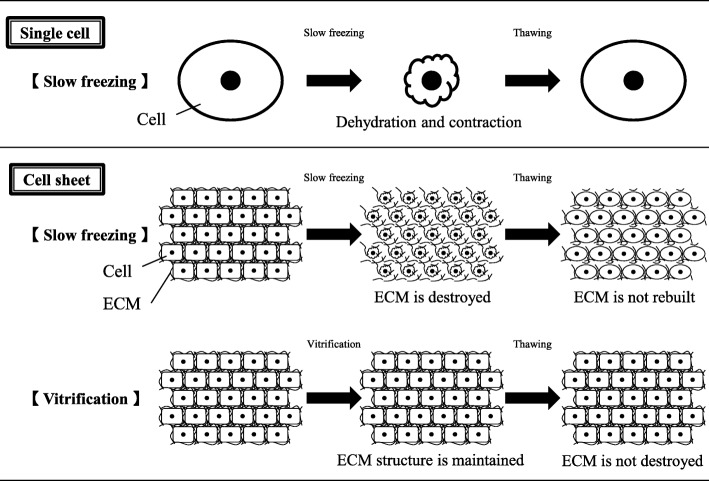
Effect of vitrification on the in vivo cardiac function of transplanted cell sheets. (Top) During slow freezing of single cells, the osmotic pressure outside the cell is high, leading to dehydration and shrinkage. If the shrinkage is excessive, the cell membrane is completely destroyed by distortion, thus causing cell death in some cases. (Middle) During slow freezing of cell sheets, a phenomenon similar to that observed in single cells occurs. It has also been reported that cell-cell junctions and the ECM of cell sheets are destroyed by ice crystal formation. (Bottom) Vitrification instantaneously brings the inside and outside of the cell to an extremely low temperature, leaving no time for intra- and extracellular water to form ice crystals. Shrinkage and membrane destruction also do not occur. Therefore, as the ECM and cell membranes are preserved, the structure is better maintained

In the established DMSO-based slow-freezing method for cell preservation [6, 7], small ice crystals form outside cells when the external temperature is below the freezing point, causing damage to the cell membrane in some cases. Additionally, the osmotic pressure outside the cell is high, leading to dehydration and shrinkage (Fig. 7). If shrinkage is excessive, the cell membrane is completely destroyed by distortion. Moreover, it has been reported that cell-cell junctions and the ECM of organs and tissues are destroyed by slow freezing (Fig. 7). It is particularly difficult to maintain homeostasis using this method for functional applications [18–20].

Vitrification avoids these problems [11, 12]. This method instantaneously brings both the inside and outside of the cell to an extremely low temperature, leaving no time for intra- and extracellular water to form ice crystals. In addition, there is no migration of water molecules between the internal and external environments of the cell; thus, shrinkage and membrane destruction do not occur (Fig. 7). Accordingly, as the ECM and cell membranes are preserved, the structure and function are better maintained.

In addition to the ECM, another crucial factor for the preservation of cell sheet structure and function is the ability to effectively maintain the activity of enzymes controlling ECM synthesis and degradation [21–26]. In general, enzyme activity tends to decline in proportion to the decrease in temperature [27]. Proteolytic enzymes may show some activity at temperatures below − 30 °C, suggesting that a cell sheet should be preserved at below − 30 °C or − 40 °C to completely stop enzyme activity. Because the storage temperature for the vitrification method is − 196 °C, we supposed that enzymes related to ECM metabolism, such as matrix metalloproteinases and tissue inhibitors of metalloproteinases, would become inactivated during vitrification. This may explain the preservation of the shape and function of the cell sheet structure when using the vitrification method.

Several cytokines secreted from cell sheets, especially HGF, play crucial roles in functional angiogenesis, which led us to examine the HGF expression level in an in vitro study. HGF expression was found to be significantly higher in the vitrification group than in the fresh group. As the cell viability was lower in the vitrification group than in the fresh group, we expected that HGF expression would accordingly show a decrease. Notably, the cell sheet preserved by vitrification instead showed higher cytokine expression levels than the fresh sheet. One possible explanation for this result is that the vitrification-preserved cell sheet might be under more stress than the fresh sheet owing to various factors, such as hypoxia or ice crystal injury. Damage or injury caused by such stress may enhance the cytokine paracrine response to protect the cells against apoptosis [28–30]. We thought that immediately after thawing, the cell sheet is in a state similar to ischemia and malnutrition, and that transcription of HGF, VEGF, SDF1 was promoted (Fig. 4a-c). Conversely, HGF showed high protein expression while VEGF and SDF1 showed low protein expression (Fig. 4d-f). We thought that HGF was translated immediately after transcription, and that protein expression was promoted. On the other hand, we thought that the translation of VEGF and SDF increased with time after transplantation.

It is known that VEGF is promoted downstream of HGF, and that VEGF and SDF are promoted downstream of HIF 1, which is increased by ischemia [31–33]. Therefore, we believe that there was a delay before VEGF and SDF reached a sufficient expression level, when compared to HGF. It was also considered that the rates of transcription and translation of each molecule were different. In any case, the cell sheet viability was maintained before and after vitrification (Fig. 1d). In the in vivo experiments, it was also observed that cardiac function improvement in the vitrification group was the same as that in the fresh group (Figs. 5 and 6). Therefore, it was considered that the levels of angiogenesis-promoting factors like VEGF and SDF increased with time after transplantation and contributed to the improvement of cardiac function.

It can be reasoned that vitrification causes some cellular damage or a decrease in cell viability because of cell shrinkage, owing to the substantial gap in osmotic pressure between the internal and external environment of the cells when the sheets are immersed in the protective vehicle. Injury might also occur during the removal of the protective vehicle after thawing. It may be possible to minimize such injury by gradually increasing the concentration of the protective vehicle during immersion of the cell sheet.

Although there was no significant difference, viability slightly decreased before and after vitrification. As the vitrification method instantly vitrifies the cells, it is unlikely that cells would be damaged by the formation of ice crystals. Instead, damage likely occurred through the formation of ice crystals when the frozen cells were thawed. To avoid this potential issue, we attempted to thaw the cryopreserved cell sheet using a hot plate but we were unable to exclude the possibility that ice crystals were generated by recrystallization [34, 35]. It may be possible to improve the cell viability by devising a more appropriate thawing method, e.g., by directly placing cell sheets in water at 37 °C to allow them to thaw rapidly.

Although cell viability in the cell sheets was lower in the vitrification group than in the fresh group, the improvement in cardiac function and angiogenesis was highly similar in both groups. Promotion of angiogenesis by cytokines, such as HGF and VEGF secreted from the cell sheet, has been demonstrated in our previous studies [1–5]. These findings suggest that the cell sheets maintain angiogenic function even after vitrification.

Myoblast cell sheets for clinical applications are generally prepared in two steps. The first step is to culture a large number of myoblasts for 2–3 weeks, followed by vitrification and preservation in liquid nitrogen (−196 °C). The second step is to prepare a cell sheet in a cell processing center where its quality is maintained for 2 days prior to the operation. Such centers are costly to establish and maintain. The method developed in the present study does not require a cell processing center in each hospital to produce cell sheets, which would substantially reduce the costs of cell sheet preparation and accordingly broaden the clinical application potential of cell sheet therapy.

Our results demonstrate the possibility of preserving the quality of cell sheets for at least 3 months after vitrification without deterioration, enabling the manufacture and dissemination of high-quality cell sheets to any hospital worldwide. Furthermore, this technology may be applicable to cell sheets derived from other cell types, such as induced pluripotent stem cell-derived cardiomyocytes. It is unlikely that the deterioration of the cell sheet will progress over time, because intracellular metabolism is nearly completely stopped at ultra-low temperatures. In the future, it will be necessary to verify the preservation for longer durations to establish the versatility of the method.

## Conclusions

Vitrification preserves the functionality and structure of scaffold-free cardiac cell-sheet constructs of human SMBs after thawing. This method may facilitate the widespread application of cell sheet therapy in clinical settings.

## Methods

### Human SMB cell culture and cell-sheet construction

Cell sheets were prepared as previously reported [36, 37]. In brief, myoblast cells were purified from 10 to 20 g of vastus medialis muscle procured from patients with heart failure (*n* = 8; age, 30–74 years [average, 56.9 years]; male:female = 6:2). The cells were suspended in skeletal muscle cell basal medium (Lonza, Walkersville, MD, USA) with fetal bovine serum (Moregate Biotech, Brisbane, Queensland, Australia), epidermal growth factor (Life Technologies, Carlsbad, CA, USA), gentamicin (Fuji Pharma, Tokyo, Japan), and amphotericin B (Thermo Fisher Scientific, Waltham, MA, USA). The cells were incubated at 37 °C in 5% carbon dioxide until the required number of cells was obtained; further passages were performed if needed. SMBs were collected and cultured on temperature-responsive culture dishes [38] (UpCell; Cell-seed, Tokyo, Japan) at a density of 1 × 10^6^ cells/cm^2^ at 37 °C for 1 day, and the cell sheets were detached from the dishes at room temperature (20–25 °C). For animal experiments, cell sheets were produced at a density of 3 × 10^6^ cells/sheet.

### Vitrification of cell-sheet constructs

SMBs were prepared as scaffold-free cell sheets using temperature-responsive culture dishes (1 × 10^6^ cells/cm^2^), as described above. The cell-sheet construct was detached from the dish at 22 °C and then immersed in protective vehicle containing 6.5 M ethylene glycol, 0.7 M sucrose, and 10% carboxyl poly-l-lysine without serum (Stem Cell Keep; Bio Verde, Kyoto, Japan) for 5 min [39]. The cell-sheet construct was covered with polypropylene mesh sheets (TiLane mesh; Medical Leaders, Tokyo, Japan), which are clinically used and served as a substrate for the cell sheet. The cell-sheet construct with the mesh was packaged between thin polyethylene films (Hybri-Bag Soft; COSMO BIO, Tokyo, Japan), which can endure ultra-low temperature. The protective vehicle and air were pressed out of the package as much as possible. The packaged cell sheet was held 1 cm above a liquid nitrogen surface for 5 min for vitrification (Fig. 1a) [17], placed in a vitrification container, and then preserved beneath the liquid nitrogen for 2 days, 7 days, 1 month, or 3 months for assessment.

### Thawing of cell-sheet constructs

A hot plate was set at 37 °C. A thermal insulation gel was placed on the hot plate and warmed for 10 min. A cell sheet was taken from the liquid nitrogen tank and placed immediately on the hot plate. The gel was placed on top of the cell sheet to thaw it rapidly. Next, each side of the package was cut, and the film was removed. The cell-sheet construct with mesh was immersed in Hanks’ balanced salt solution containing Ca^2+^ and Mg^2+^ to remove the protective vehicle. After removal of the mesh, the cell sheet was analyzed.

### Cell viability assay

Cell viability was evaluated by cell counting using Trypan blue. In brief, cell sheets were immersed in 500 μL of TrypLE Select (Thermo Fisher Scientific) and incubated at 37 °C for 15 min. The suspension was centrifuged at 400×*g* for 5 min and the supernatant was removed. Single cells were washed with 500 μL of Hanks’ balanced salt solution containing Ca^2+^ and Mg^2+^ (HBSS(+)). The suspension was centrifuged at 400×*g* for 5 min and the supernatant was removed. Cells were suspended in 100 μL HBSS (+) and stained with Trypan blue. The number of viable cells and the number of dead cells were counted using a microscope to calculate the viability.

### Animal experiments

Male nude rats (F344/NJcl-*rnu/rnu* (CLEA Japan, Inc.); 8–10 weeks of age) underwent left coronary artery ligation as previously described [37, 40]. The rats were anesthetized by inhalation of isoflurane and placed on a respirator during surgery to maintain ventilation. After 2 weeks, echocardiography was performed to evaluate the extent of MI, and rats with an EF greater than 30% and less than 50% were used as MI model rats. The MI model rats were randomly divided into three treatment groups: a fresh group (cell sheets used without further treatment, *n* = 11), vitrification group (n = 11), and sham-operation group (*n* = 10). The cell sheets were transplanted onto the infarct area in the fresh and vitrification groups. In the sham-operation group, rats were subjected to thoracotomy, and the chest was closed without transplantation. The effect of the transplantation of various sheets on cardiac function was evaluated by echocardiography, including estimates of the EF, FS, ΔLVDd, ΔLVDs, and ΔIVSd at 2 and 4 weeks after transplantation. The rats were anesthetized by inhalation of isoflurane to alleviate suffering and the hearts were quickly procured to determine the extent of fibrosis of the left ventricle (%), the length of the minor axis of the cells (μm), and the number of microvessels (per mm^2^).

### Histological analysis

Cell sheets and heart ventricles of nude rats were immersion-fixed in 4% paraformaldehyde, embedded in paraffin, and cut into 5-μm sections using a microtome. The sections were stained with HE or SR and assessed by optical microscopy [36, 41]. Metamorph software (Molecular Devices, Sunnyvale, CA, USA) was used to separate the SR-stained and non-stained myocardium and to quantitatively measure each area. The sections were immunohistologically labeled with polyclonal anti-von Willebrand factor antibody (1:100) (DAKO, Glostrup, Denmark) at 4 °C overnight, and visualized using the Universal LSAB2 System HRP Kit (DAKO), which is an automated immunostaining system based on the LSAB Lepto-streptavidin-biotin-peroxidase method [36, 41, 42].

### Immunohistochemistry

Cell sheets were fixed with 4% paraformaldehyde, which was then replaced with 30% sucrose. The cell sheets were embedded using optimal cutting temperature compound (Sakura Finetek Japan, Tokyo, Japan) and cut into 5-μm sections using a cryostat. The sections were incubated with Protein Block (Agilent Technologies, Santa Clara, CA, USA) and then labeled with primary antibodies against fibronectin (1:50), collagen I (1:50), N-cadherin (1:100), and integrin α5 (1:100) (all from Abcam, Cambridge, UK) at 4 °C overnight, followed by incubation with fluorescence-conjugated secondary antibodies for visualization, including Alexa Fluor 555-conjugated goat anti-rabbit IgG (1:100) and Alexa Fluor 488-conjugated phalloidin for F-actin staining (1:50) (all from Life Technologies) at 37 °C for 2 h. Nuclei were counterstained with 4′,6-diamidino-2-phenylindole dihydrochloride (1:100) (Life Technologies) at room temperature (20–25 °C) for 10 min. The sections were assessed by confocal laser-scanning microscopy (FV10i-DOC; Olympus Corporation, Tokyo, Japan).

### Apoptosis analysis

Frozen sections of cell sheets were immunostained using an Annexin 5 antibody (1:100) (Abcam) as described above. The sections were assessed using a fluorescence microscope (BZ-9000; KEYENCE, Tokyo, Japan). The level of apoptosis was calculated by the ratio of Annexin 5-positive cells to all cells.

### Transmission electron microscopy (TEM)

Cell sheets were fixed with 1/2 strength Karnovsky’s fixative at 4 °C overnight for primary fixation, and then with 2% osmium tetroxide for 2 h at 4 °C for post-fixation. The cell sheets were dehydrated with ethanol and embedded with epoxy resin. Ultrathin sections (90–120 nm) were prepared using an ultra-microtome (EM UC7; Leica Microsystems GmbH, Wetzlar, Germany), and stained with uranyl acetate and lead citrate on a grid for electron microscopy. Ultrathin sections were observed by TEM (H-7650; Hitachi, Tokyo, Japan).

### Western blot analysis

Cell sheets were transferred to 200 μL of lysis buffer and denatured using NuPAGE LDS sample buffer (4×) (Invitrogen, Carlsbad, CA, USA) at 70 °C for 10 min. Equal amounts of protein were loaded onto a gel for sodium dodecyl sulfate-polyacrylamide gel electrophoresis, and then transferred from the gel onto a polyvinylidene fluoride membrane (GE Healthcare UK, Buckinghamshire, UK). The membrane was coated with blocking buffer for 20 min and reacted with the primary antibody (fibronectin, 1:500; collagen I, 1:5000; N-cadherin, 1:400; integrin α5, 1:1000; and glyceraldehyde-3-phosphate dehydrogenase [GAPDH], 1:2000; all from Abcam) at 4 °C overnight. The membranes were then reacted with horseradish peroxidase-linked secondary antibodies (ECL anti-rabbit IgG, 1:2000; and ECL anti-mouse IgG, 1:2000; GE Healthcare UK) at room temperature (20–25 °C) for 1 h. The membranes were visualized using the ECL Prime western blotting detection reagent (GE Healthcare UK), and images were acquired using the ChemiDoc MP Imaging System (BioRad, Hercules, CA, USA). All bands were quantified by densitometry and normalized to GAPDH levels.

### Enzyme-linked immunosorbent assay (ELISA)

The concentrations of VEGF, HGF, and SDF-1α in supernatants were detected by ELISA using commercially available ELISA kits (R&D Systems, Minneapolis, MN, USA). Optical densities were determined using a microplate reader (BioTek Instruments) at 450 nm, with wavelength correction set to 540 nm.

### Real-time reverse transcription-polymerase chain reaction (qRT-PCR)

Total RNA was extracted using the PureLink RNA Mini Kit (Life Technologies) and reverse-transcribed into cDNA using Omniscript reverse transcriptase (Qiagen, Hilden, Germany). qPCR was performed using the TaqMan Fast Advance Master Mix (Life Technologies) with the following gene primers (all from Thermo Fisher Scientific): *VEGF* (Hs00900055_m1), *HGF* (Hs00300159_m1), *SDF-1* (Hs03676656_mH), *mt*-*ND1* (Hs02596873_s1), *SDHA* (Hs00188166_m1), *mt*-*ATP6* (Hs02596862_g1), and *GAPDH* (Hs03929097_g1). All assays were performed using the Viia7 real-time PCR system (Life Technologies), and the average gene expression level was normalized to that of *GAPDH* [43].

### Statistical analysis

Data were statistically analyzed in Microsoft Office Excel (Redmond, WA, USA). The *t*-test was used for comparisons between 2 groups. *p* < 0.05 was considered statistically significant.

## Abbreviations

DMSO: Dimethyl sulfoxide
ECM: Extracellular matrix
EF: Ejection fraction
ELISA: Enzyme-linked immunosorbent assay
FS: Fractional shortening (FS)
HBSS(+): Hanks’ balanced salt solution containing Ca^2+^ and Mg^2+^
HE: Hematoxylin and eosin
*HGF*: Hepatocyte growth factor
LV: Left ventricular
MI: Myocardial infarction
*mt*-*ATP6*: Mitochondrial-encoded ATP synthase 6
*mt*-*ND1*: Mitochondrial-encoded NADH dehydrogenase 1
qRT-PCR: Quantitative reverse transcription polymerase chain reaction (qRT-PCR)
*SDF1*: Stromal cell-derived factor-1
*SDHA*: Succinate dehydrogenase complex, subunit A
SMB: Skeletal myoblast
SR: Sirius Red
TEM: Transmission electron microscopy
*VEGF*: Vascular endothelial growth factor
ΔIVSd: Difference in interventricular septum dimension before and after treatment
ΔLVDd: Difference in left ventricular end-diastolic dimension before and after treatment
ΔLVDs: Difference in left ventricular end-systolic dimension before and after treatment
